# Mn- and Yb-Doped BaTiO_3_-(Na_0.5_Bi_0.5_)TiO_3_ Ferroelectric Relaxor with Low Dielectric Loss

**DOI:** 10.3390/ma16062229

**Published:** 2023-03-10

**Authors:** Dong-Yun Gui, Xiao-Yong Ma, Hu-Die Yuan, Chun-Hai Wang

**Affiliations:** 1 College of Materials Science and Engineering, Xi’an University of Architecture and Technology, Xi’an 710055, China; 2 State Key Laboratory of Solidification Processing, Northwestern Polytechnical University, Xi’an 710072, China

**Keywords:** BaTiO_3_, relaxor ferroelectrics, complex impedance and modulus, dielectric loss

## Abstract

In this work, a Mn-and Yb-doped BaTiO_3_-(Na_0.5_Bi_0.5_)TiO_3_ ferroelectric relaxor was designed and prepared. The effects of Mn on the microstructures, dielectric and electrical properties of the ceramics were investigated. The X-ray structural analysis shows a perovskite structure. The SEM images show the homogeneous microstructure of ceramics with an average grain size of about 1 μm. The temperature-dependent permittivity shows relaxor characteristics as Mn-doped. Mn at a low level (x ≤ 0.005) is beneficial for low dielectric loss and high resistivity. The maximum resistivity of ≥3 × 10^12^ Ω cm and minimum dielectric loss of ≤0.06 can be achieved at *x* ≤ 0.005. The resistivity of the ceramics follows the Arrhenius law with activation energy decreasing from ~1.31 to 1.01 eV as *x* increases. With lower Mn dopant, oxygen vacancies and charge carrier concentration partially decrease with Mn doping, which is helpful to improve the insulation resistance and decrease the dielectric loss.

## 1. Introduction

The interesting physical properties of relaxor ferroelectrics that exhibit diffusion during phase transitions have gained considerable attention, as they have practical potential for applications such as electrostrictive components [1], PTCRs (positive temperature coefficient resistors) [2,3], multilayer ceramics capacitors (MLCCs) [4] and multicaloric applications [5]. Classic ferroelectrics can be transformed into relaxor ferroelectrics (RFEs) by means of a doping strategy, such as BaTiO_3_ (BT)-based [6], (Na_0.5_ Bi_0.5_)TiO_3_ (NBT)-based [7,8] and other traditional ferroelectric systems [9,10].

The effects of doping with various oxides in classic ferroelectric materials have been extensively studied by many researchers. It was found that the Curie temperature (TC) becomes higher as NBT is added in BT-based ceramics [11]. For example, the Curie temperature increases from 125 to 156 °C with 8 mol% NBT added into BT, making it possible to satisfy the MLCC criterions [12]. The trivalent rare earth ions and manganese ions were found to be effective for the improvement of the temperature-dependent permittivity of BaTiO_3_ dielectrics [13,14]. However, the addition of the trivalent (La^3+^, Y^3+^) [15,16,17,18] donor dopants cause an increase in room-temperature conductivity. Ding et al. reported that the resistance of BT ceramics decreases with Y doping and increases dramatically with Mn doping. Gong et al. studied the resistance degradation and the conduction mechanism of Mn-doped nano-BaTiO_3_ ceramics. They found that the resistivity reliability characteristics greatly depended on the Mn ions. Incorporating 0.3 mol% of manganese has a positive impact on enhancing the reliability of BaTiO_3_-based ceramics, which is a crucial factor for the application of multilayer ceramic capacitors (MLCCs). Mn-doping at the Ti site in BT has been extensively studied by several groups [19,20,21,22], and it was found that the Mn acts as an acceptor, which improves the electrical resistivity near the Curie temperature. Furthermore, Mn^2+^ or Mn^4+^ ions as acceptor dopants were found to be located at the grain boundaries to enhance the PTC effect [23,24]. In summary, it was found that the rare earth (Ln) oxides are effective dopants in BT-based ferroelectric materials and lead to a broader *ε_r_*′-*T* dispersing curve. However, ferroelectric relaxors based on Ln-doped BT often exhibit low resistivity and high dielectric loss [14,25,26], which results in high energy consumption in devices made from these materials. Since the dielectric loss in ferroelectric relaxors is mainly caused by electric conduction, samples with higher resistivity generally exhibit lower dielectric loss. Therefore, it is expected that co-doping Mn and Ln could modify both the temperature-dependent permittivity dispersion and dielectric loss (resistivity) of BT-NBT ferroelectric relaxors, leading to the development of ferroelectric relaxors with lower dielectric loss.

In this paper, Mn-Yb co-doped BT-NBT ceramic samples were designed and synthesized. Using X-ray diffraction, SEM, LCR meter and AC impedance measurements, we analyzed the microstructure, dielectric relaxation behavior and electrical properties, particularly the resistivity and dielectric loss behavior. The results indicate that the co-doping of Mn-Yb in BT-NBT leads to the production of a low-loss ferroelectric relaxor.

## 2. Materials and Methods

The conventional solid-state reaction method was used to prepare the 0.92BT-0.08NBT-0.02Yb-*x*Mn (*x* = 0.0025, 0.005, 0.01, 0.015, 0.02) (BT-NBT-Yb-*x*Mn) ceramic samples. After pre-synthesizing NBT powder from reagent-grade Na_2_CO_3_, Bi_2_O_3_ and TiO_2_ calcined at 800 °C for 2 h, BT and NBT powders were mixed in a mole ratio of 0.92:0.08. The slurries were dried and calcined at 900 °C for 2 h, resulting in the formation of a BT-NBT solid solution, and then mixed with 0.02 mol Yb_2_O_3_ and *x* mol MnCO_3_ in ethanol, and ball-milled with zirconium media for 24 h. After drying, the powders were added to 4 wt.% polyvinyl alcohol (PVA) as a binder and pressed into pellets. The compacts were sintered at a temperature range of 1230–1280 °C for 2 h, followed by meticulous polishing of the resulting ceramics.

To determine the phase composition and lattice parameters, X-ray powder diffraction (XRD) was carried out using Cu Kα radiation (X’Pert PRO, PANalytical, Almelo, The Netherlands). The microstructures of the ceramics were examined using SEM (JSM-5610LV, JEOL, Tokyo, Japan). The pellets with dimensions of 10 mm (diameter) and 1 mm (thickness) were coated with Ag paste as electrodes on both sides for dielectric measurement. Temperature-dependent capacitance and dielectric loss were measured with an LCR meter (HP E4980A, Palo Alto, Santa Clara, CA, USA) at 1 KHz, 10 KHz, 100 KHz and 200 KHz, from room temperature to 200 °C. For the measurement of electrical properties, the Pt paste was pasted on ceramic samples as electrodes. Impedance spectra were measured for the ceramics in the frequency range of 10^−2^ to 10^6^ Hz, from room temperature to 700 °C using a Solartron 1260 impedance/grain-phase analyzer and 1296 dielectric interface. Impedance results were analyzed using ZView software(v3.0a).

## 3. Results and Discussions

### 3.1. Structural and Microstructural Study

**Figure 1:**
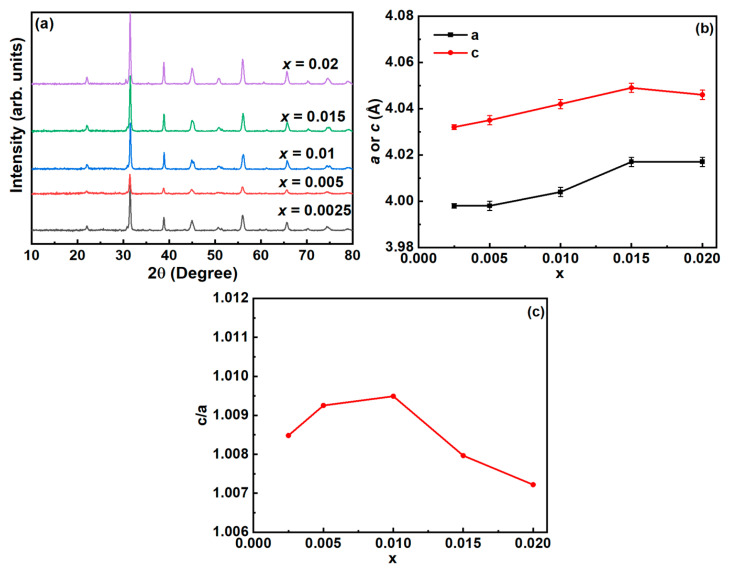
(**a**) XRD patterns of BT-NBT-Yb-*x*Mn samples; (**b**) cell parameters *a* and *c* calculated from the XRD data; (**c**) the tetragonality *c*/*a* from Pawley refinements of all samples.

The phase of the samples was determined by X-ray diffraction analysis, and the corresponding patterns are presented in Figure 1a for the BT-NBT-Yb-*x*Mn ceramics. The samples gave similar XRD patterns, which match the BT tetragonal phase [27], and no obvious impurity reflections were observed. A single-phase perovskite structure with tetragonal symmetry was exhibited with the sample *x* = 0.015, showing a distinct peak splitting of (002) and (200) peak at around 45° in the 2*θ* range representing a normal tetragonal structure, but the peaks overlap each other in other samples representing a pseudo-cubic structure, which means it is hard to recognize the tetragonal phase or cubic one from the peak splitting. The lattice parameters and tetragonality utilizing the Pawley refinements program in the as prepared samples are shown in Figure 1b,c. Both the *a* and *c* increase with the increase of *x* and reach the maximum as *x* = 0.015, which means that a solid solution was formed at *x* ≤ 0.015. Referring to the ionic radii of Mn and Ti^4+^ in the octahedra [coordination number *CN* = 6, *R*(Mn^2+^) = 0.83Å, *R*(Mn^3+^) = 0.58Å, *R*(Mn^4+^) = 0.53Å, *R*(Ti^4+^) = 0.61Å)] [28], it can be inferred that the Mn in the solid solution should be Mn^2+^ and B-O bonds (B = Mn, Ti) become longer as Mn^2+^ is added. The tetragonality (*c*/*a*) of the samples gradually increases from 1.0085 for *x* = 0.0025 to 1.0095 for *x* = 0.01, and then decreases to 1.0072 for *x* = 0.02. Notably, the tetragonality (*c*/*a*) values are quite close to the ideal cubic condition with *c*/*a* = 1.000, leading to the definition of the samples as pseudo-cubic phase. As the ionic radii of Mn and Ti^4+^ differ, Mn causes more significant distortions in [BO_6_] (B = Mn or Ti) octahedra than in the original BT-NBT-Yb. This is expected to affect the diffusion in the phase transition (dielectric relaxing) of the samples [29].

**Figure 2:**
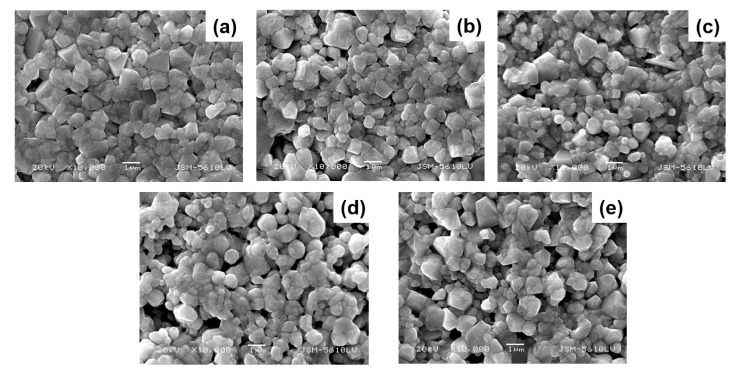
SEM images of fractured BT-NBT-Yb-*x*Mn ceramics samples. (**a**) *x* = 0.0025, (**b**) *x* = 0.005, (**c**) *x* = 0.01, (**d**) *x* = 0.015, (**e**) *x* = 0.02.

Figure 2a–e shows the SEM images of fractured surfaces of sintered BT-NBT-Yb-*x*Mn ceramics. All specimens show similar microstructures with an average grain size of about 1 µm. It is clear that the Mn does not affect the average grain size of BT-NBT-Yb-*x*Mn ceramics.

### 3.2. Dielectric Relaxation

**Figure 3:**
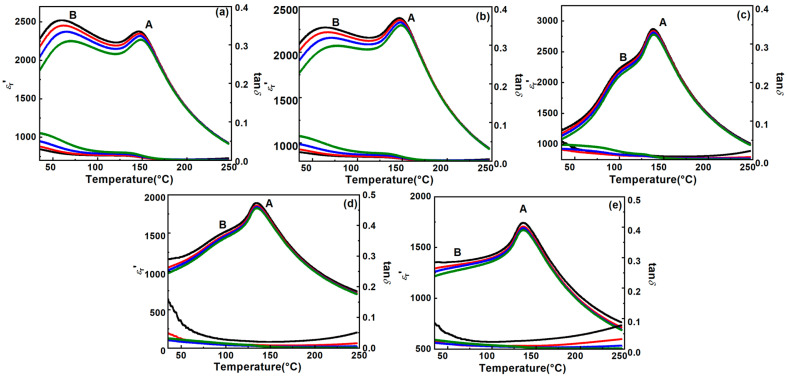
The temperature-dependent permittivity (*ε_r_′*) and the dielectric loss (tan*δ*) of the BT-NBT-Yb-*x*Mn samples were measured at frequencies *f_1_* = 1 KHz, *f_2_* = 10 KHz, *f_3_* = 100 KHz and *f_4_* = 200 KHz. (**a**) *x* = 0.0025 (**b**) *x* = 0.005 (**c**) *x* = 0.01 (**d**) *x* = 0.015 (**e**) *x* = 0.02; A, B denote different peaks of *ε_r_*′-*T*.

The temperature-dependent permittivity and the dielectric loss (tanδ) of BT-NBT-Yb-*x*Mn from LCR measurements are shown in Figure 3. As Mn was doped into the samples, the dielectric loss (tanδ) of all BT-NBT-Yb-*x*Mn samples are all lower than 0.1. The dielectric loss is obviously lower than that of samples without Mn [26], which generally give a tanδ of ~0.3. Sample BT-NBT-Yb-0.01Mn gives the low dielectric loss tanδ < 0.05, and samples with *x* ≤ 0.01 are close to each other. The temperature-dependent permittivity curve is strongly affected by the composition. There are two clear characteristic dielectric peaks. For the permittivity at 1 KHz, the permittivity of peak A increases from 2379 at 147 °C for *x* = 0.0025 to 2875 at 138 °C for *x* = 0.01, then decreases to 1742 at 135 °C for *x* = 0.02. The permittivity of peak B decreases from an obvious band at *x* ≤ 0.005 to a weak shoulder (*x* = 0.01), and then to a plateau (*x* = 0.02). The permittivity of peak B decreases with increasing frequency and the corresponding temperature shifts to higher temperatures as *x* = 0.01, which is a typical relaxor behavior of ceramic. On the other hand, the permittivity of peak B shows a very broad peak for *x* = 0.02. The broadening peak observed in the temperature dependence of the permittivity of BT-NBT-Yb-*x*Mn ceramics was confirmed to be a relaxor, which exhibits a slow phase transition along with a strong dispersion of the temperature of maximum dielectric permittivity against frequency.

According to the XRD results, the tetragonality changes with the composition and should be the origin of classical ferroelectric behavior, in which a higher tetragonality *c*/*a* should give a larger permittivity. Combing the XRD and permittivity results, the permittivity is expected to show a maximum at *x* = 0.01. The change of permittivity of peak A is consistent with the change of tetragonality very well, which means that the permittivity of peak A is dominated by the main phase. However, the permittivity of peak B is almost independent of the tetragonality *c*/*a*, which means that one is controlled by the other factors (microstructure).

Several factors have been identified in the literature that play an important role in the variation of temperature-dependent permittivity (diffused phase transition). These include cation ordering and internal stresses. Alkathy et al. reported that in Bi- and Li-co-doped BaTiO_3_, grain size distribution is a significant factor affecting the diffuse character of the dielectric permittivity peak. As the concentrations of Bi and Li increased, the average grain size decreased, and diffusivity increased [30]. Armstrong et al. showed that the permittivity of BaTiO_3_ modified with 2 wt% ZrO_2_ resulted in a suppressed ferroelectric transition region. They attributed this to the unique microstructure of core–shell grains and mismatch internal stress between the core and shell regions, leading to a flat temperature-dependent permittivity [31]. Furthermore, Yung et al. investigated how stress affects the dielectric temperature properties of cerium-modified barium titanate. By using TEM, they observed three different inhomogeneous regions, including grain core, grain shell and gradient regions, and they explained how the dielectric temperature characteristics are related to the regions and internal stress [32]. As the average grain size of BT-NBT-Yb-*x*Mn ceramics is not affected by the doping of Mn from SEM analysis, the change of temperature-dependent permittivity in BT-NBT-Yb-*x*Mn should be caused by the inhomogeneous regions of grain core, grain shell, gradient ones and internal stress. As indicated by SEM analysis, the doping of Mn does not affect the average grain size of BT-NBT-Yb-*x*Mn ceramics. Therefore, the changes in the temperature-dependent permittivity of BT-NBT-Yb-*x*Mn can be attributed to inhomogeneous regions with the grain core, grain shell and gradient ones, as well as internal stress.

### 3.3. Impedance Analysis

**Figure 4:**
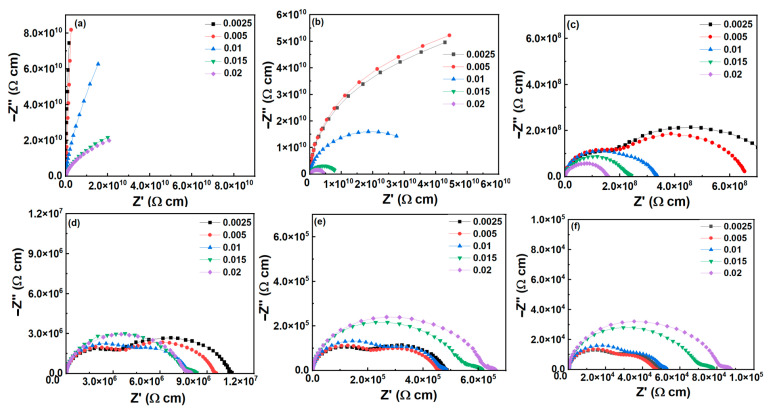
Nyquist plots of BT-NBT-Yb-*x*Mn samples at various temperatures. (**a**) 100 °C, (**b**) 200 °C, (**c**) 300 °C, (**d**) 400 °C, (**e**) 500 °C, (**f**) 600 °C.

The impedance Z* plots for all samples in a wide temperature (100–600 °C) are measured and shown in Figure 4. At low temperatures (≤200 °C), each sample exhibits a single or partial semicircle, indicating a response attributed to the grain. This suggests that the grain provides high resistance, which is advantageous for minimizing dielectric loss associated with low leakage current. There are two overlapping arcs observed at *T* ≥ 300 °C for samples with *x* ≤ 0.01, which are from the grain and grain boundaries of the ceramics. However, there was only grain arc response observed for samples with *x* ≥ 0.015.

**Figure 5:**
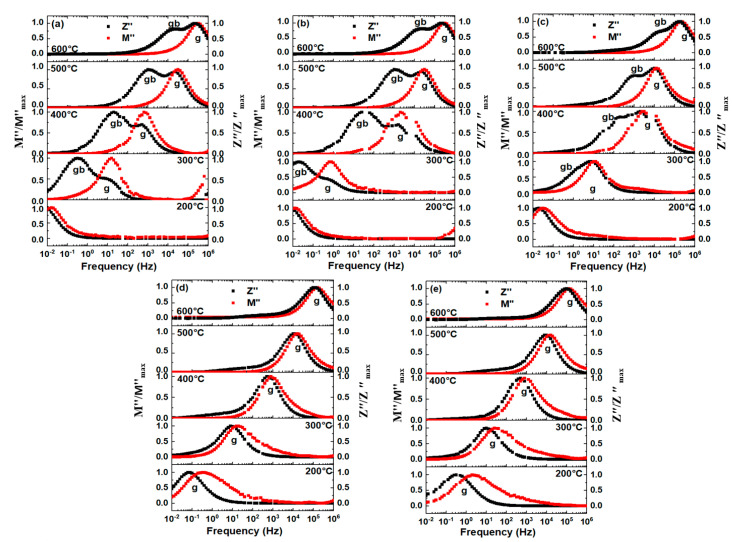
The normalized *−Z*″ and *M*″ of BT-NBT-Yb-*x*Mn at 200–600 °C. (**a**) *x* = 0.0025, (**b**) *x* = 0.005, (**c**) *x* = 0.01, (**d**) *x* = 0.015, (**e**) *x* = 0.02. g: grain; gb: grain boundary.

The normalized *Z*″ and *M*″ against frequencies at selected temperatures are plotted in Figure 5. With the increase in temperature, the maximum *Z*″*_max_* and *M*″*_max_* shift to the higher frequency. The similar frequency of the *Z*″*_max_* and *M*″*_max_* peaks indicate that the relaxation describes the same response. The *Z*″ shows two relaxation peaks for the lower Mn-doped compounds (*x* ≤ 0.01, Figure 5a–c) in which the peak at a lower frequency and the other at a higher frequency are from the relaxation process of grains and grain boundaries, respectively. However, in the samples with *x* = 0.015 and *x* = 0.02, both the *Z*″ and *M*″ show one peak at a close frequency, which is dominated by the grain response.

**Figure 6:**
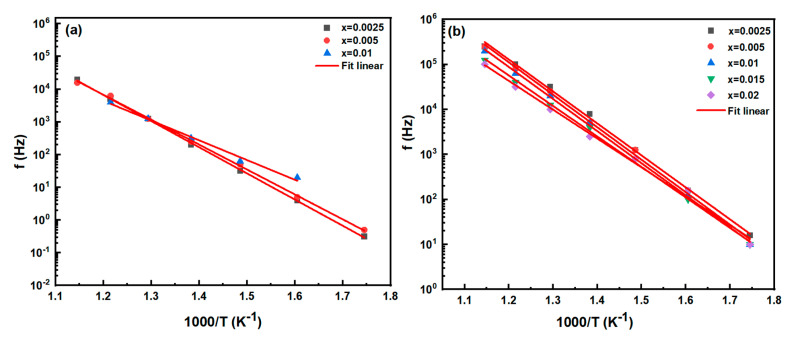
Temperature-dependent frequency of *Z*″*_max_* and *M*″*_max_* of BT-NBT-Yb-*x*Mn (**a**) grain boundary and (**b**) grain.

The temperature-dependent *Z*″ and *M*″ peak frequencies of samples are presented in Figure 6. Using the Arrhenius equation, the activation energy of both the grain and grain boundaries in BT-NBT-Yb-*x*Mn ceramics were determined through calculations based on the imaginary part of the modulus and impedance.

$$\omega_{p}\left(T\right)=2\pi f_{p}\left(T\right)=Aexp\left(\frac{-E_{a}}{kT}\right)$$

where *A* is a constant, *E_a_* is the activation energy, *k* is the Boltzmann constant and *T* is the absolute temperature. The activation energies calculated from the linear fit are listed in Table 1. As the doping of Mn increases, the activation energy decreases, particularly for the *x* = 0.015 and *x* = 0.02. This should be caused by the chemical defect concentration increase with the increase of Mn.

### 3.4. Frequency Dependent ε_r_′ and ε_r_″

**Figure 7:**
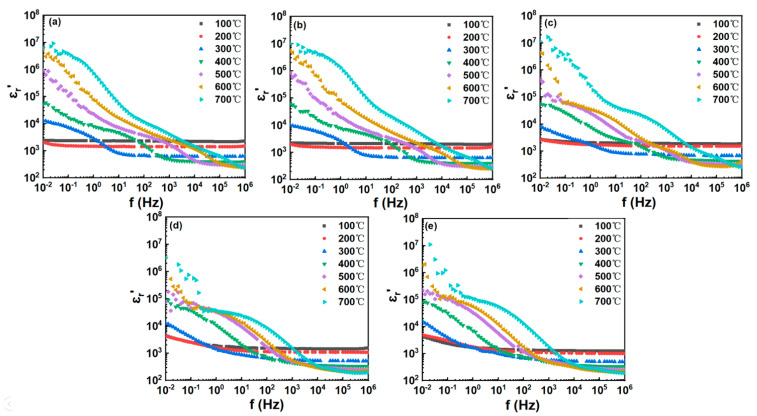
Frequency-dependent permittivity (*ε_r_*′) of BT-NBT-Yb-*x*Mn with Pt electrode at various temperatures. (**a**) *x* = 0.0025, (**b**) *x* = 0.005, (**c**) *x* = 0.01, (**d**) *x* = 0.015, (**e**) *x* = 0.02.

The real part of permittivity (*ε_r_*′) as a function of frequency at different temperatures of the BT-NBT-Yb-*x*Mn ceramic is shown in Figure 7a–e. At very low frequencies, the *ε_r_*′ increases for all samples as the temperatures increase. At temperatures 200–700 °C, there are broad dispersed steps with relatively high permittivity observed in the low frequency region and the step shifts to a higher frequency with the increase of temperature. This behavior is associated with a frequency-dependent orientational polarization. For *x* = 0.0025, high dielectric permittivity (at ≤10 Hz) is observed at high temperatures. The *ε_r_*′ at 0.01 Hz is almost two magnitudes of orders larger than that at 10 Hz. This should be caused by extrinsic contributions such as space charge localization at the grain boundaries [33]. The permittivity *ε_r_*′ is almost independent of frequency at low temperatures (≤200 °C) with plateaus for all samples, except slight increases at low frequency (f < 1 Hz) for *x* = 0.015 and *x* = 0.02 samples.

For high frequencies, permittivity *ε_r_*′ decreases with the temperature and reaches a plateau. Referring to the impedance analysis, the relative permittivity observed at 10 Hz ≤ f ≤ 10^6^ Hz is caused by the dielectric relaxation from the grain and/or grain boundaries. In addition, the temperature-dependent permittivity (Figure 3) measured from LCR meter is coincident with that from impedance measurements (frequency-dependent curves in Figure 7). 

**Figure 8:**
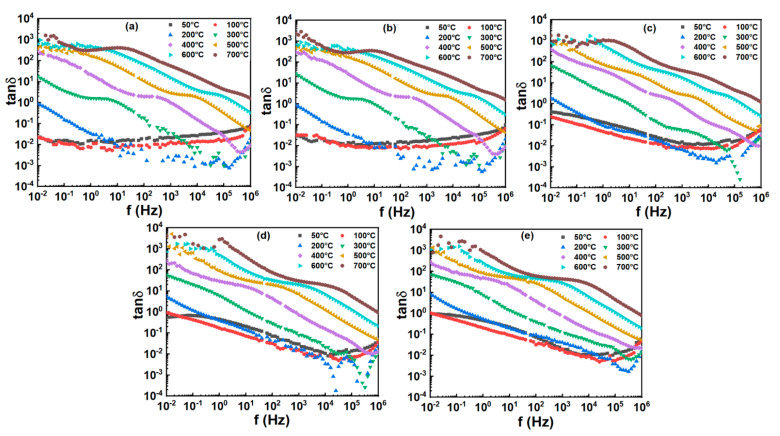
Frequency dependent of dielectric loss (tan*δ*) of BT-NBT-Yb-*x*Mn with Pt electrode measured at various temperatures. (**a**) *x* = 0.0025, (**b**) *x* = 0.005, (**c**) *x* = 0.01, (**d**) *x* = 0.015, (**e**) *x* = 0.02.

The frequency-dependent tan*δ* at different temperatures of BT-NBT-Yb-*x*Mn ceramics are shown in Figure 8. The tan*δ* shows a plateau at temperatures below 100 °C, which is less than 0.06 from 0.01 Hz to 10^6^ Hz and remains almost unchanged with *x* ≤ 0.005. At 50 °C, the tan*δ* at 1 KHz are 0.02, 0.02, 0.02, 0.03, 0.02 for *x* = 0.0025, 0.005, 0.01, 0.015 and 0.02, respectively. The samples show lower tan*δ* (≤0.06) as *x* ≤ 0.01, and tan*δ* increases with *x* > 0.01. As temperature increases, the samples show a higher tan*δ* step at a low frequency, and the step shifts to a higher frequency. For example, as *x* = 0.005, the maximum of tan*δ* located at the frequency of 3.98 Hz, 251 Hz, 6309 Hz, 50,119 Hz and 39811 Hz at 300 °C, 400 °C, 500 °C, 600 °C and 700 °C, respectively. This can be described as RC resonance at a lower frequency and smaller polarization at a high frequency.

### 3.5. Resistivity

**Figure 9:**
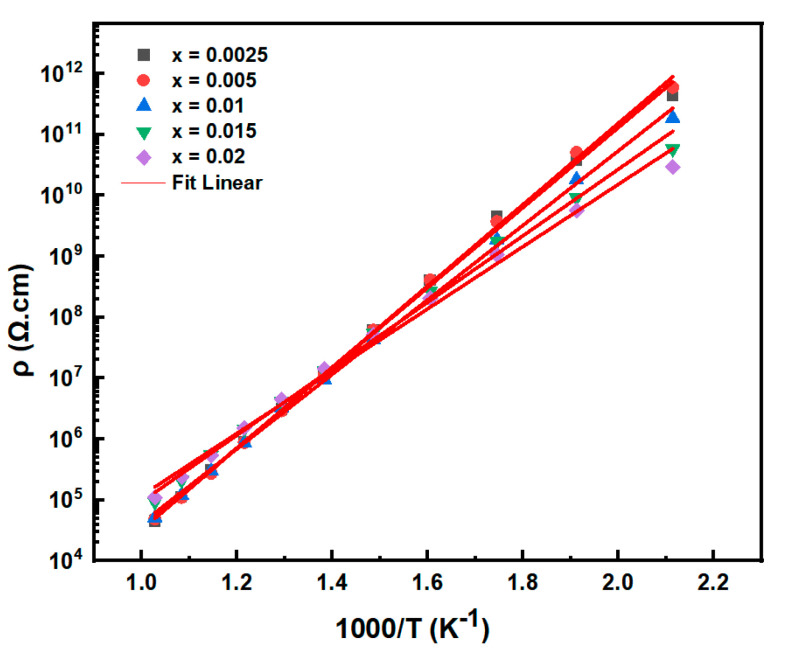
Temperature-dependent resistivity (*ρ*) of BT-NBT-Yb-*x*Mn.

The temperature-dependent resistivity of BT-NBT-Yb-*x*Mn samples are shown in Figure 9. At room temperature, the resistivity calculated from impedance analysis is ≥3 × 10^12^ Ω cm, ≥3 × 10^12^ Ω cm, 1.61 × 10^11^ Ω cm, 3.39 × 10^10^ Ω cm and 1.61 × 10^10^ Ω cm for *x* = 0.0025, 0.005, 0.01, 0.015 and 0.02, respectively. At 200 °C, the resistivity is 4.37 × 10^11^ Ω cm, 5.88 × 10^11^ Ω cm, 1.86 × 10^11^ Ω cm, 5.77 × 10^10^ Ω cm and 2.91 × 10^10^ Ω cm for *x* = 0.0025, 0.005, 0.01, 0.015 and 0.02, respectively. The resistivity shows a maximum at *x* = 0.005 and decreases with the increase of Mn. The resistivity of samples with *x* = 0.005 is about two times higher than that of samples with the BT-NBT-Yb sample. Thus, the doping of Mn shows an obvious increase in resistivity. The activation energy (*Ea*) calculated is 1.29 eV, 1.31 eV, 1.16 eV, 1.08 eV and 1.01 eV for *x* = 0.0025, 0.005, 0.01, 0.015 and 0.02, respectively. The activation energies decrease with the increase of Mn, and the samples with *x* ≤ 0.005 give the highest activation energy (*Ea*).

As is known, the properties of oxide materials, particularly their electrical resistance, are significantly influenced by the concentration and distribution of oxygen vacancies. It is noteworthy that oxygen vacancies are the defects most commonly found in oxide materials [34]. It was found that *Ea* for all samples was about 1eV, which is very close to the activation energy of oxygen vacancies [35]. In a BT-NBT-Yb-*x*Mn system, the Mn should be on the Ti^4+^ site as an acceptor in the perovskite solid solution of BT-NBT as the ionic radius of Mn^2+^ (0.83 Å as *CN* = 6 and 0.96 Å as *CN* = 8) is closer to Ti^4+^ (0.61 Å as *CN* = 6) than Ba^2+^ (1.42 Å as *CN* = 8 and 1.61 Å as *CN* = 12) [28]. The neighboring acceptor–oxygen vacancy pairs were formed to maintain electro-neutralization; Mn ions can reduce the oxygen vacancies in the perovskite. With a small amount of Mn addition, it is helpful to improve the insulation resistance of the ceramics. These ceramics are, therefore, well suited for applications as dielectric materials. With higher dopant concentration (*x* ≥ 0.015), Mn induces the formation of various new defects, which causes a decrease in resistivity [13].

## 4. Conclusions

A single perovskite-type structure was achieved for BT-NBT-Yb-*x*Mn ceramics prepared via solid-state reaction. The Nyquist plot, complex impedance, complex modulus, resistivity and activation energy of charge carriers were analyzed to study the microstructure–electrical property relationships. The temperature-dependent permittivity shows dielectric relaxations. The small amount of Mn (*x* ≤ 0.005) causes an obvious decrease in dielectric loss. The BT-NBT-Yb-*x*Mn with *x* ≤ 0.005 gave the lowest dielectric loss of tan*δ* ~0.06. The resistivity of BT-NBT-Yb-*x*Mn was >3 × 10^12^ Ω cm as *x* ≤ 0.005 and decreased to 1.61 × 10^10^ Ω cm as *x* = 0.02. The activation energy of charge carriers calculated from the temperature dependence of resistivity decreased from 1.29 eV to 1.01 eV as the x increased from 0.0025 to 0.02.

## Figures and Tables

**Table 1:** Activation energy obtained from the *Z*″ and *M*″ of BT-NBT-Yb-*x*Mn.

Sample	*Ea* (Grain, eV)	*Ea* (Grain Boundary, eV)
*x* = 0.0025	1.41	1.58
*x* = 0.005	1.42	1.51
*x* = 0.01	1.41	1.20
*x* = 0.015	1.34	
*x* = 0.02	1.27	

## Data Availability

Data will be made available on request.
